# Cost-effectiveness of modified diagnostic strategy to safely rule-out pulmonary embolism in the emergency department: a non-inferiority cluster crossover randomized trial (MODIGLIA-NI)

**DOI:** 10.1186/s12873-023-00910-x

**Published:** 2023-11-29

**Authors:** Arnaud Nze Ossima, Bibi Fabiola Ngaleu Siaha, Maroua Mimouni, Nadia Mezaour, Meryl Darlington, Laurence Berard, Marine Cachanado, Tabassome Simon, Yonathan Freund, Isabelle Durand-Zaleski

**Affiliations:** 1 Paris Health Economics and Health Services Research Unit, URC Eco IdF, AP-HP, Hotel Dieu, 1 Place du Parvis Notre Dame, Paris, 75004 France; 2 Département de Pharmacologie Clinique and Plateforme de Recherche Clinique de l’Est Parisien, URCEST-CRC-CRB), Assistance Publique–Hôpitaux de Paris, Sorbonne Université, Hôpital St Antoine, Paris, France; 3 Service des Urgences, Hôpital Pitié–Salpêtrière, AP-HP, Paris, France; 4 URCEco Hôpital de l’Hôtel Dieu, Paris, F-75004 France

**Keywords:** Pulmonary embolism, Emergency, Cost-effectiveness

## Abstract

**Background:**

The aim of this trial-based economic evaluation was to assess the incremental costs and cost-effectiveness of the modified diagnostic strategy combining the YEARS rule and age-adjusted D-dimer threshold compared with the control (which used the age-adjusted D-dimer threshold only) for the diagnosis of pulmonary embolism (PE) in the Emergency Department (ED).

**Methods:**

Economic evaluation from a healthcare system perspective alongside a non-inferiority, crossover, and cluster-randomized trial conducted in 16 EDs in France and two in Spain with three months of follow-up. The primary endpoint was the additional cost of a patient without failure of the diagnostic strategy, defined as venous thromboembolism (VTE) diagnosis at 3months after exclusion of PE during the initial ED visit. Mean differences in 3-month failure and costs were estimated using separate generalized linear-regression mixed models, adjusted for strategy type, period, and the interaction between strategy and period as fixed effects and the hospital as a random effect. The incremental cost-effectiveness ratio (ICER) was obtained by dividing the incremental costs by the incremental frequency of VTE.

**Results:**

Of the 1,414 included patients, 1,217 (86%) were analyzed in the per-protocol analysis (648 in the intervention group and 623 in the control group). At three months, there were no statistically significant differences in total costs (€-46; 95% CI: €-93 to €0.2), and the failure rate was non inferior in the intervention group (-0.64%, one-sided 97.5% CI: -∞ to 0.21%, non-inferiority margin 1.5%) between groups. The point estimate of the incremental cost-effectiveness ratio (ICER) indicating that each undetected VTE averted in the intervention group is associated with cost savings of €7,142 in comparison with the control group. There was a 93% probability that the intervention was dominant. Similar results were found in the as randomized population.

**Conclusions:**

Given the observed cost decrease of borderline significance, and according to the 95% confidence ellipses, the intervention strategy has a potential to lead to cost savings as a result of a reduction in the use of chest imaging and of the number of undetected VTE averted. Policy-makers should investigate how these monetary benefits can be distributed across stakeholders.

**Clinicaltrials:**

Trial registration number ClinicalTrials.gov Identifier: NCT04032769; July 25, 2019.

**Supplementary Information:**

The online version contains supplementary material available at 10.1186/s12873-023-00910-x.

## Introduction

Pulmonary embolism (PE) and venous thromboembolism (VTE) are an important health and economic burden, with estimated direct and indirect costs of about 10,000€ per confirmed case of PE, and the loss of over one healthy year in Europe [1]. Medical direct costs in the USA have been estimated at least 10 billion yearly, most of which are hospital related [2, 3].

The optimal diagnostic strategy for patients with suspected pulmonary embolism remains under debate. The usual diagnostic strategy implemented consists of initially estimating the prior probability from clinical information, followed by a D-dimer test (in patients with a low clinical probability), and, if the D-dimer level is above a certain threshold, chest imaging (computed tomography pulmonary angiography [CTPA] or pulmonary ventilation/perfusion scintigraphy [V/Q scan]). This strategy presents with an approximate diagnostic yield of 10% [4].

In order to safely reduce the use of CTPA in emergency departments (ED) and the additional costs involved, a modified diagnostic strategy, which combines the YEARS rule (clinical criteria that elevates the D-dimer threshold for prescribing CPTA) with the pulmonary embolism rule-out criteria (PERC) rule and the age-adjusted D-dimer threshold was tested in the MODIGLIA-NI study [5, 6]. The use of PERC rule has become standard in many EDs and was considered routine practice in the participating centers. This is also suggested by the European Society of Cardiology guidelines, especially in the low prevalence population, and by a recent review on PE diagnostic [7, 8]. The primary clinical outcome was failure of the diagnostic strategy, defined as venous thromboembolism (VTE) diagnosed at 3-months after exclusion of PE during the initial emergency department (ED) visit and the non-inferiority was confirmed [3].

In this study, we estimated the cost-effectiveness of the YEARS rule and age-adjusted D-dimer threshold (intervention) compared with age-adjusted D-dimer threshold (control). This economic evaluation was conducted alongside the trial with three-months of follow-up.

## Patients and methods

### Design of the MODIGLIA-NI trial and population

This trial-based economic evaluation was based on MODIGLIA-NI, a non-inferiority, crossover, cluster-randomized trial conducted in 16 EDs in France and two in Spain. The study protocol has been previously published [4]. In summary, each ED was randomized to a diagnostic strategy to rule out PE either with age-adjusted D-dimer threshold (control) for four months, followed, after a two-month washout period, by the YEARS rule and age-adjusted D-dimer threshold (intervention) for four months, or in reverse order. The randomization ratio was 1:1. Randomization was stratified by country and ED size (small versus large defined as > or < than 50,000 patients per year) [8] (Figure S1 in the Supplementary material). Patients who had a low clinical risk of PE not excluded by the PERC rule or a subjective clinical intermediate risk of PE were included. Patients with a negative PERC rule (low probability and PERS score = 0) were not included.

The primary outcome in the efficacy trial was the percentage of failure of the diagnostic strategy (undetected VTE), defined as Venous thromboembolism (VTE) diagnosis at 3 months after exclusion of PE during the initial ED visit.

The secondary end points were: chest imaging (CTPA or V˙ /Q˙ scan) ordered by ED physicians, ED length of stay, hospital admission following the ED visit, anticoagulant administration, all-cause mortality, and all-cause readmissions at 3 months.

### Cost-effectiveness analysis

We followed a detailed health economics analysis plan developed before the end of the trial and last modified before the freezing of the trial database. We chose a cost-effectiveness analysis instead of a cost minimization procedure, with a non-inferiority margin of 1.35%, as described in the published study protocol [9]. This economic evaluation of the innovative diagnostic strategy of PE followed the recommendations from the French national health authority and the reporting follows the CHEERS statement for single trial-based studies [10]. All data necessary for the economic analysis, including those covering medical resource use and major events, were collected prospectively within the MODIGLIA-NI trial.

The primary medical outcome was the percentage of patients without undetected VTE averted (defined as venous thromboembolism (VTE) diagnosis at 3 months after exclusion of PE during the initial ED visit). The economic endpoint was the incremental cost-effectiveness ratio (ICER), expressed as the difference in costs divided by the difference in patients without cost per undetected VTE averted (i.e. patients correctly diagnosed), between the intervention and control strategies. Total costs were estimated from inclusion (the visit to ED) and over a three-month follow-up period. Given the non-inferiority hypothesis, the ICER was initially planned to estimate the small health loss (acceptable increase in diagnostic failure) for a reduction in cost.

Due to the short duration of this study, costs and outcomes were not discounted. We adopted a healthcare system perspective with cost expressed in Euros (€), in 2022 prices [11].

### Resource use and costs

Resource use was collected prospectively in the electronic Case Record Form (eCRF) developed for the MODIGLIA-NI trial on the Cleanweb™ ® software, completed at the initial ED visit and for all hospitalizations at a three-months horizon. During the initial ED visit, data related to laboratory tests including D-dimer use, CTPA use, ventilation–perfusion [V̇/Q̇] scan use, and technical charges (for equipment and maintenance) were collected. The total length of stay of VTE related hospitalizations and discharge information (using the severity adjusted diagnostic related groups or DRGs) were extracted from the hospital information system for the index admission and subsequent admissions during the three-month follow-up period.

The cost of laboratory tests was estimated from the French national tariffs of D-dimer testing, CTPA, [V̇/Q̇] scan and technical charges (Table S1 in the Supplementary material). The hospitalization costs were estimated from the French National Cost Study that collects yearly data at the patient level from a sample of public or not-for-profit hospitals and estimates an average cost of production per DRG with 95% confidence intervals [12]. These costs include medical and related procedures, personnel (medical and non medical) costs, treatments (except specific expensive drugs), food and accommodation, and investment costs. Only direct medical costs were considered [12]. The DRG-specific costs were adjusted for actual length of stay of the study patients. Calculation of hospital costs in Spain is done differently by region. We decide to standardize Spanish resource use on French costs, using the information collected on the length of stay, procedures and tests. Table S1 in the supplementary material presents the calculation method, data sources and type of unit costs used.

### Sample size calculation

The sample size calculation was based upon clinical hypotheses, with a noninferiority margin set at 1.35%, an anticipated failure rate of 0.5% in the control group, the 2-sided α risk set at 5% and β set at 20%. The cluster design effect was estimated 1.37. Assuming that 5% of patients would not be evaluable, with 18 EDs and 2 periods per ED, 1234 patients were needed.

### Statistical analysis

Analysis was conducted in both the per-protocol (PP) and as randomized populations. The per-protocol population excluded patients who did not meet all inclusion and non-inclusion criteria, were not treated using the strategy allocated to the ED, had a missing value for the primary end point, or had any other major protocol deviation identified during the data review just before the database was frozen. In the as randomized population, patients with missing values for the primary endpoint were classified as having a VTE.

Baseline patient characteristics were described overall and for each group using the number (percentage) for categorical variables and the mean (SD). The arithmetic mean is the usual summary statistic to consider the total cost from the budgetary perspective.

Differences in mean costs and frequency of VTE were estimated using separate generalized linear-regression mixed models, with gamma distribution and log link for costs and a Bernoulli distribution (logit link) for frequency, both of which were estimated controlling for diagnostic strategy, period, strategy-by-period interaction as fixed effects, and cluster at the hospital-level as a random effect. We then calculated incremental cost-effectiveness ratio (ICER) by dividing the incremental costs by the incremental effects. The ICER indicates the cost per undetected VTE averted.

The uncertainty surrounding these point estimates was examined using a two-stage non-parametric bootstrapping technique with 1,000 replications [14]. This method explicitly accounts for the correlation and clustering in the hierarchical cost and effect data. The 2,5th and the 97,5th centile of the 1,000 bootstrap replications form the 95% uncertainty interval of the differences in costs, effects, and ICER. The 1000 ICERs were plotted on cost-effectiveness plane. In a cost-effectiveness plane, the horizontal axis displays the difference in effects and the vertical axis displays the difference in costs. The results of the bootstrap replications can fall into one of four quadrants: northeast quadrant (more cost and more effects); southeast quadrant (less cost and more effects); southwest quadrant (less cost and less effects); northwest quadrant (more cost and less effects).

### Missing data

Missing data were managed differently between the two countries. For French re-hospitalization costs related to VTE, one patient out of the six with a VTE event had missing DRG information, so the cost was imputed by the mean re-hospitalization cost of the strategy group. For the Spanish centers, cost data at the index date was not collected for all 36 patients so a multiple imputation was performed by group strategy using a chained model with 25 iterations, regressed on the baseline complete covariates: sociodemographic characteristics (age, sex, body mass index), clinical characteristics, period, and clustering. The imputation model was estimated using predictive mean matching.

All analyses were performed with R freeware (version 4.1.2).

## Results

### Patient characteristics at baseline

**Fig. 1:**
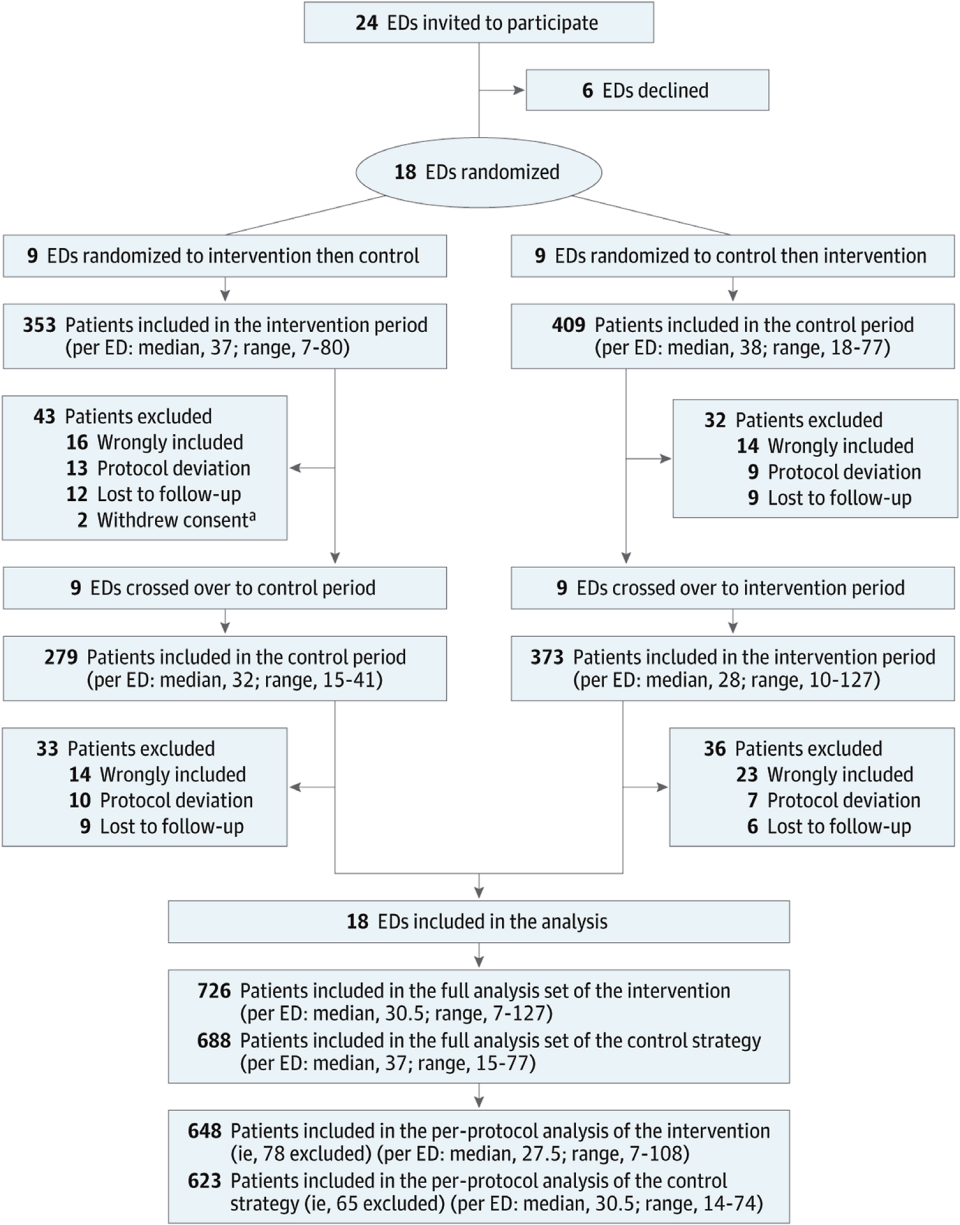
Patients flow diagram (ClinicalTrials.gov, NCT04032769. Registered on 24 July 2019)

**Fig. 2:**
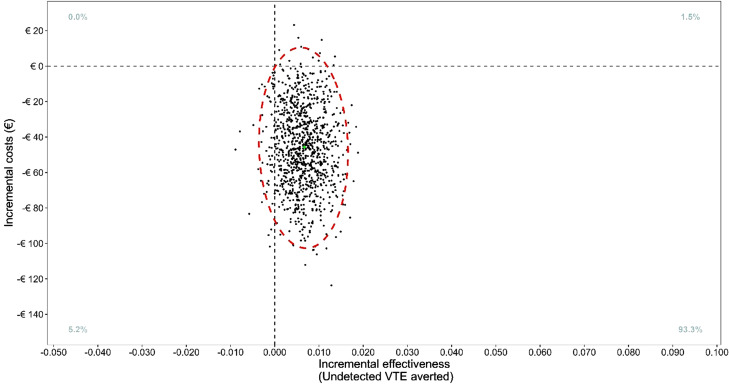
Bootstrap distribution of 1000 ICERs in (€ / undetected VTE averted) at three months (The vertical red line indicates the non-inferiority margin)

Detailed information on the trial’s design, inclusion and exclusion criteria, population characteristics, and the diagnosis of VTE, results have been published. Briefly, 1,414 patients were randomized to either intervention strategy group (n = 726) or control strategy group (n = 688) in 16 emergency departments in France and 2 in Spain (Figure S2; Table S2 in Supplementary material). The trial included 1414 patients in the randomized population (Fig. 1; Table S4). After exclusion of 67 further ineligible patients and 39 patients with major protocol deviations, 1,271 were included in the per-protocol analysis (648 in the intervention group and 623 in the control group) (Fig. 2). There was no significant difference in the characteristics of patients at baseline as shown in Table S2 in supplementary material. The mean (SD) age was 55 (19) years and 58% were female. PE was diagnosed in the ED in 100 patients (7.1%): 54 (7.4%) and 46 (6.7%) in the intervention and control groups, respectively (Table S2 for the per-protocol and S3 for the as-randomized populations). At 3 months, VTE was diagnosed in 1 patient in the intervention group (0.15% [95%CI, 0.0–0.86%]) vs. 5 patients in the control group (0.80% [95%CI, 0.26%to 1.86%]) (difference expressed as intervention minus control and adjusted for periods as fixed effects and cluster as a random effect, − 0.64% [1-sided 97.5%CI, −_ to 0.21%], within the non-inferiority margin). The intervention group was associated with lower use of chest imaging compared with a conventional strategy, with the absolute difference between the 2 groups being 9% (30.4%vs 40.0%; adjusted difference, − 8.7% [95%CI, − 13.8% to − 3.5%]). The median ED length of stay was 6.0 h (IQR, 4.0–8.0) vs. 6.0 h (IQR, 5.0–9.0) (adjusted difference, − 1.6 h [95% CI, − 2.4 to − 0.9]).

### Resource use and costs

D-dimer testing was performed in the entire the per-protocol population and 29.8% of the intervention group had a CPTA test compared to 40.1% in the control group. Data on whether or not a hospital admission occurred was unavailable for 32 (2.5%) patients. Six patients were hospitalized for VTE at three months (one in the intervention group and five in the control group); detailed hospitalization codes are presented in table S4. Total costs per patient were €297 in the intervention group and €349 in the control group. The adjusted difference of the costs between intervention and control groups was €-46 but this difference was not statistically significant ([95% CI, €-93; €0.2]) (Table 1).

**Table 1:** Summary of resource use and costs (at inclusion, at follow-up). Figures are presented either in n (%) or mean ± sd

Variable	Groups		Difference	
	**Intervention group** **(n = 648)**	**Control group** **(n = 623)**	**Adjusted for periods as fixed effects and cluster as a random effect** **ΔCosts or ΔEffects (95% CI)**	**Unadjusted** **ΔCosts or ΔEffects (95% CI)**
**Per-protocol population**				
**Index Admission**				
**1. ER visit**				
YEARS score = 0*	515 (75.5)	-	-	-
CTPA or V/Q lung scan done**	193 (29.8)	250 (40.1)	-	-
**2. Hospitalization**				
**Admitted from the ED**	143 (22.1)	160 (25.68)	-	-
**Average length of stay index (Days)**	1.87 (± 5.68)	1.97 (± 5.35)	-	-
**Rehospitalization: Follow-up period at 3 months**				
**VTE at 3 months**	1 (0.15)	5 (0.80)	− 0.64 [97.5% 1-sided CI, -∞ to 0.21]	− 0.65 [97.5% 1-sided CI, -∞ to 0.17]
**Mean cost per patient**				
**Emergency room**	116 (± 39)	123 (± 46)	-	-7 [95% CI, -12.8; -3.2]
**Index hospitalization**	181 (± 351)	211 (± 374)	-	-30 [95% CI, -68; 19]
**Hospitalization admission at 3 months related to VTE**	0.3 (± 7)	15.2 (± 194)	-	-15 [95% CI, -37; -0.5]
**Total costs**	297 (± 364)	349(± 466)	-46 [95% CI, -93; 0.2]	-52 [95% CI, -102; 0.4]

* YEARS score ranges from zero to three, 1 point per item: PE is the most likely diagnostic, hemoptysis, and clinical sign of deep vein thrombosis.

** One patient in the control group had CTPA and [V˙/Q˙] scan.

### Cost-effectiveness analysis

In the PP population, the results of the cost-effectiveness analysis for undetected VTE averted are presented in Table 1. The ICER was 7,142 indicating that each undetected VTE averted in the intervention group is associated with cost savings of €7,142 in comparison with the control group. Most of the bootstrapped ICER replications and the confidence ellipse were in the southeast quadrant (Fig. 1). Furthermore, 93% of the bootstrap replications were within the southeast quadrant. Altogether, these data indicate that intervention is a cost-effective strategy and has a high probability of being a dominant strategy.

The cost-effectiveness results for the as randomized population are presented in Table S5 (Supplementary material). The ICER was 8,982 indicating that each undetected VTE averted in the intervention group is associated with cost savings of €8,982 in comparison with the control group. Similarly, most of the confidence ellipse was in the southeast quadrant with 85% of the bootstrap replications. Diagnostic intervention thus appears to have been a cost-effective strategy, and can be considered a dominant strategy with a high probability (Figure S2 in the Supplementary material).

## Discussion

In this within-trial economic evaluation of the use of the YEARS criteria combined with the age-adjusted D-dimer threshold compared to the standard diagnostic strategy to rule out pulmonary embolism in the emergency department, we found a high probability that combining YEARS and age-adjusted D-dimers is less costly and non inferior to the control with age-adjusted D-dimers alone. The majority of bootstrap replications (93% of cases) in the CE planes showed that intervention strategy had a high probability of being a dominant strategy, both cost saving and outcome improving.

Our results show that the point estimates of cost and effect-difference between the intervention group and the control group was not statistically significant, although total costs and failure rate were lower in the intervention strategy than in the control strategy. The strict protocol for patient management in the ED made it unlikely to allow large differences in resource use, although the use of chest imaging was 9% lower in the intervention strategy. The largest contributor to the cost difference was the hospital admission following ED workup, but it only amounted to 30€ because of the low admission rate. Both strategies were successful at ruling out PE without false negatives, which was an ethical requirement of the protocol and explains the small difference in re admission rates and costs.

In addition, the sample size was based on the primary outcome of the presence of VTE at 3 months, and therefore was insufficiently powerful to detect relevant cost differences. However, the health economics literature is clear that the decision to estimate incremental cost-effectiveness should not be based on separate and sequential hypothesis tests concerning cost and effect differences, but should instead consider the joint density of cost and effect differences. The results here bear out this recommendation, showing that modest cost and effect differences considered jointly produce a high probability that the intervention will be less costly and more effective.

To our knowledge, our study is the first formal cost-effectiveness evaluation of a strategy that combines the YEARS rule and the age-adjusted D-dimer cutoff. However, a number of previous cost-effectiveness analyses of diagnostic strategies including D-dimer to exclude PE have been published. A systematic review in 2022 [15] identified twelve studies, concluding that these diagnostic strategies including D-dimer were cost-effective compared with strategies without. It is difficult to compare with these studies, because the inclusion criteria differed and the efficacy outcomes were not failure to diagnose VTE, but either mortality, life years or QALYs. The only first formal cost-effectiveness evaluation of the Age-adjusted D-dimer threshold was performed with QALYs as criteria of effectiveness. With a health care system perspective, the authors were concluded that the use of the age-adjusted D-dimer was associated with an average cost reduction of US$33.4. Only study have compared the age-adjusted D-dimer cutoff strategy to the YEARS strategy in ED visits [16], however without an evaluation of its effectiveness and without hospitalization costs. With French health care costs, authors estimated an average decrease by €123. The larger cost savings in this study, compared with our findings on ED visit, largely arose from the costs of the ED capacity who were considerable (€108 savings).

These results were derived from a pragmatic trial with a large sample size and 18 participating hospitals, which allowed prospective collection of relevant cost and effect data, and enabled the evaluation of the intervention’s cost-effectiveness. The randomization ensured a high internal validity of the results. The pragmatic design, the large number of participating centers with broad inclusion criteria and adherence to current guidelines in the control group suggest that the external validity is also good. Although the cost are based on French data, they would be close to those in other Western European countries, albeit not in the USA.

This study presents several limitations, which include the limitations of the trial listed in the princeps publication [4]. An other limitation concerns the unavailability hospitalization data (only 2.5%). Although this is an acceptable rate of missing data, we tried to account for this by applying multiple imputation for missing data the amount of incomplete data.

## Conclusion

Cost-effectiveness analyses primarily aim to provide complementary information that can help a decision-maker to choose one strategy over another. In this context, the present study provides crucial information about the cost and cost-effectiveness of intervention strategy. Given the observed cost decrease of borderline significance, and according to the 95% confidence ellipses, the intervention strategy has a potential to lead to cost savings as a result of a reduction in the use of chest imaging and of the number of undetected VTE averted. Policy-makers should investigate how these monetary benefits can be distributed across stakeholders.

### Electronic supplementary material

Below is the link to the electronic supplementary material.

**Supplementary Material 1:** Calculation methods and unit costs for the cost efectivenesss analysis

## Data Availability

Yes. Data types: Deidentified participant data. How to access data: All the study data will be provided for review upon reasonable request to the corresponding author. When available: With publication Supporting Documents. Document types: None Additional Information Who can access the data: researchers whose proposed use of the data has been approved. Types of analyses: IPD Meta analysis of PE diagnostic studies. Mechanisms of data availability: After approval of the sponsor and the corresponding author.
