# Supplementary material for: Cost-effectiveness of modified diagnostic strategy to safely rule-out pulmonary embolism in the emergency department: a non-inferiority cluster crossover randomized trial (MODIGLIA-NI)
Source: BMC Emerg Med. 2023 Nov 29;23:140. doi: 10.1186/s12873-023-00910-x (PMC10687836; doi:10.1186/s12873-023-00910-x)
Supplement: Supplementary file 1 — Supplementary Material 1: Calculation methods and unit costs for the cost efectivenesss analysis [file 12873_2023_910_MOESM1_ESM.docx]

**Supplementary material**

Figure S1: Diagnostic Strategy for Pulmonary Embolism (PE) (ClinicalTrials.gov, NCT04032769. Registered on 24 July 2019)


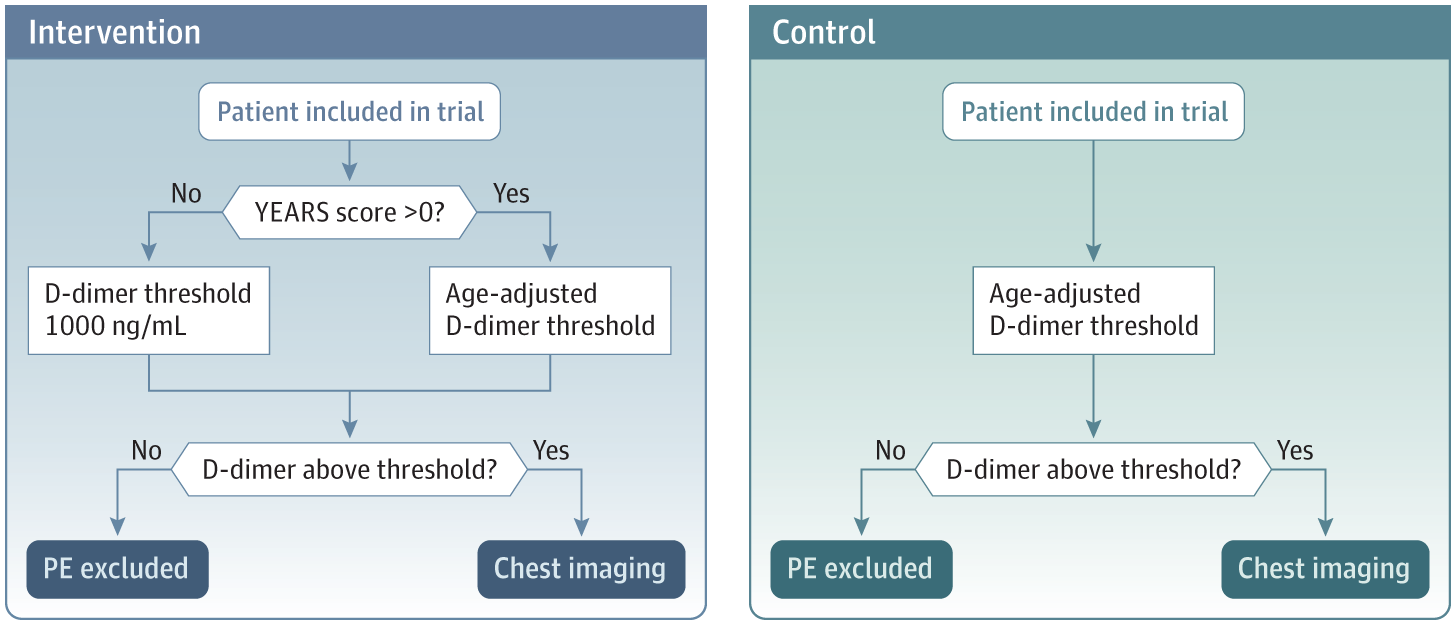


Table S1: Method of calculating the costs of resource use and unit costs

|  | **Calculation method** | **Sources of data** | **Unit costs** |
| --- | --- | --- | --- |
| **ED visit** | Cost of laboratory tests = fixed ED cost (€16.20) + cost of D-dimer (€29.26)+ cost of CTPA (or V/Q scan) (if imaging done) (€357.29)+ cost of technical package (if imaging done) (€71.38) | The acts carried out in the ED are collected from e-CRF | Report to the Senate on the emergency departments (17) |
| **All-cause Index hospitalization and re-hospitalization related to PE** | Cost of hospital stay = (cost of the stay for the DRG / mean length of stay observed in DRG) x DS | The duration of stay (DS) of each patient and the Diagnosis Related Group (DRG) are collected from the local PMSI (hospital billing database). | The cost of the stay is valued using DRGs from the national production cost study (ENCC) 2018. (11) |
| **Code (if applicable)** | **Label** | **Cost/tariff (euros)** | **Source** |
| Not applicable | Fixed ER cost | 80 | *Assurance Maladie* (18) Hausfater, 2020 (19), |
| 1022 | D-dimer | 16.2 | *Table Nationale de codage de Biologie* (20) |
| ECQH010 | CTPA | 29.26 (25.27 + 15.8% - medical act realised by a radiologist) | *Classification Commune des Actes Médicaux* (CCAM) (18) |
| GFQL006 | V/Q scan | 357.29 | *Classification Commune des Actes Médicaux* (CCAM) (18) |
| - | Technical package (ref activity class 1) | 71.38 | *Assurance Maladie* (18) |
| - | Technical package (ref activity class 3) | 29.63 | *Assurance Maladie* (18) |
| STF | Intensive care supplement | 402.51 | ENCC, 2018 (15) |
| REA | Resuscitation supplement | 804.07 | ENCC, 2018 (15) |
| SRC | Continuous monitoring supplement | 322.01 | ENCC, 2018 (15) |

1. Les urgences hospitalières, miroir des dysfonctionnements de notre système de santé [Internet]. [cited Jan 16, 2023]. Available on: https://www.senat.fr/rap/r16-685/r16-6857.html
2. CCAM en ligne - CCAM [Internet]. [cited Jan 16, 2023]. Available on: https://www.ameli.fr/accueil-de-la-ccam/index.php
3. Hausfater P, Hajage D, Bulsei J, Canavaggio P, Lafourcade A, Paquet AL, et al. Impact of Point-of-care Testing on Length of Stay of Patients in the Emergency Department: A Cluster-randomized Controlled Study. Academic Emergency Medicine. 2020;27(10):974‑83.
4. National fee schedule for biology. Accessed 27/07/2023[TNB : Liste (cnamts.fr)](http://www.codage.ext.cnamts.fr/cgi/nabm/cgi-liste?p_code_nabm=&p_nom_court=D+DIMERES&p_nb=3&p_site=AMELI)

Table S2: Patients characteristics (Per-protocol population)

| **Patient Characteristics, No.** | **Intervention group**  **N = 648** | **Control group**  **N = 623** |
| --- | --- | --- |
| Age (yrs.) | 54.64 ± 19.1 | 55.70 ± 19.8 |
| Gender: Female | 354 (54.6) | 392 (62.9) |
| Congestive or ischemic heart disease, | 57 (8.85) | 69 (11.08) |
| Chronic respiratory insufficiency | 33 (5.1) | 38 (6.1) |
| Chronic renal failure (estimated clearance < 30 ml/min) | 12 (1.8) | 11 (1.7) |
| Stroke, | 13 (2) | 13 (2) |
| Cancer: Active, | 25 (4) | 40 (6) |
| Cured < 12 months | 10 (1.56) | 13 (2.1) |
| Past Thrombo-embolic event (PE/DVT) | 54 (8.3) | 50 (8.0) |
| Chest pain | 464 (71.6) | 455 (73.1) |
| Dyspnea | 358 (55.2) | 356 (57.2) |
| Syncope | 74 (11.4) | 67 (10.7) |
| Respiratory rate (breaths per minute) | 18.8 ± 4.65 | 19.2 ± 4.84 |
| Heart rate (BPM) | 91.1 ± 20.1 | 92.4 ± 21 |
| Temperature (°C) | 36.7 ± 0.6 | 36.8 ± 0.65 |
| Systolic blood pressure (mmHg) | 140 ± 20.66 | 140 ± 21.8 |
| SaO2 (%) | 97.1 ± 2.65 | 97 ± 2.93 |
| Pain on lower limb deep venous palpation | 18 (2.8) | 28 (4.5) |
| Lower limb unilateral edema | 6 (1) | 10 (1.6) |
| Immobilization or surgery within 1 month | 33 (5) | 22 (4) |
| Exogen estrogen use (pilule/THS) | 66 (18.9) | 55 (14.0) |
| Anticoagulant therapy given in emergency room | 15 (2.3) | 19 (3.0) |
| DDMIER < seuil age | 387 (59.7) | 373 (59.8) |
| DDMIER < 1000 | 492 (75.9) | 451 (72.4) |
| PE (pulmonary embolism) is the most likely diagnosis | 53 (8.1) | 43 (6.9) |
| In case of PE, specify type  Isolated Subsegmental  Lobar  Segmental  Subsegmental | 1 (1.9)  24 (45.3)  18 (33.96)  7 (13.21) | 2 (2.33)  17 (39.53)  20 (46.51)  2 (4.65) |
| Patient outcome  Hospitalization  Transfer to another hospital  Return to home | 126 (19.4)  17 (2.6)  504 (77.8) | 150 (24.1)  10 (1.6)  463 (74.3) |

Figures are presented either in n (%) or mean±sd.

Table S3: Patient characteristics (As randomized population)

| **Patient Characteristics, No.** | **Intervention group**  **N = 726** | **Control group**  **N = 688** |
| --- | --- | --- |
| Age (yrs.), mean ± SD | 53.9 ± 19 | 55.4 ± 19.6 |
| Gender: Female, No. (%) | 397 (54.7) | 426 (61.9) |
| Congestive or ischemic heart disease, No. (%) | 63 (8.7) | 78 (11.3) |
| Chronic respiratory insufficiency, No. (%) | 38 (5.3) | 40 (5.8) |
| Chronic renal failure (estimated clearance < 30 ml/min), No. (%) | 12 (1.7) | 13 (1.9) |
| Stroke, No. (%) | 15 (2.1) | 15 (2.1) |
| Cancer: Active, No. (%) | 28 (3.9) | 43 (6.2) |
| Cured < 12 months, No. (%) | 12 (1.7) | 14 (2.0) |
| Past Thrombo-embolic event (PE/DVT), No. (%) | 56 (7.7) | 54 (7.9) |
| Chest pain, No. (%) | 525 (72.3) | 494 (71.9) |
| Dyspnea, No. (%) | 391 (53.9) | 396 (57.6) |
| Syncope, No. (%) | 79 (10.9) | 72 (10.4) |
| Respiratory rate (breaths per minute), mean ± SD | 18.8 ± 4.61 | 19.3 ± 4.91 |
| Heart rate (BPM), mean ± SD | 90.9 ± 20.1 | 92.1 ± 20.9 |
| Temperature (°C), mean ± SD | 36.7 ± 0.6 | 36.8 ± 0.64 |
| Systolic blood pressure (mmHg), mean ± SD | 140 ± 20.6 | 140 ± 22.5 |
| SaO2 (%), mean ± SD | 97.2 ± 2.63 | 97 ± 2.98 |
| Pain on lower limb deep venous palpation, No. (%) | 19 (2.6) | 32 (4.6) |
| Lower limb unilateral edema, No. (%) | 7 (1) | 14 (2) |
| Immobilization or surgery within 1 month, No. (%) | 35 (4.8) | 25 (3.6) |
| Exogen estrogen use (pilule/THS), No. (%) | 68 (17.3) | 56 (13.2) |
| Anticoagulant therapy given in emergency room, No. (%) | 16 (2.2) | 23 (3.3) |
| DDMIER < seuil age, No. (%) | 432 (59.5) | 410 (59.7) |
| DDMIER < 1000, No. (%) | 562 (77.4) | 501 (73) |
| PE (pulmonary embolism) is the most likely diagnosis, n (%) | 54 (7.4) | 46 (6.7) |
| In case of PE, specify type, n (%)  Isolated Subsegmental  Lobar  Segmental  Subsegmental | 1 (1.85)  24 (44.44)  18 (33.33)  8 (14.81) | 3 (6.52)  17 (36.96)  20 (43.48)  2 (4.35) |
| ED discharge disposition, n (%)  Hospital admission  Transfer to another hospital  Discharged home | 140 (19.3)  17 (2.3)  568 (78.2) | 166 (24.1)  12 (1.7)  510 (74.1) |
| Patients hospitalized during follow-up, n (%) | 85 (11.7) | 99 (14.4) |

Table S4: Diagnostic related groups at admission

| **DRG** | **Label** | **Mean length-of-stay (days)** | **Costs (€)** |
| --- | --- | --- | --- |
| 04M101 | Pulmonary embolism, level 1 | 5.6 | 3,003 |
| 04M102 | Pulmonary embolism, level 2 | 7.3 | 3,759 |
| 04M103 | Pulmonary embolism, level 3 | 11.8 | 5,725 |
| 04M104 | Pulmonary embolism, level 4 | 17.3 | 10,563 |
| 04M10T | Pulmonary embolism, very short duration | 1.1 | 1,097 |
| 04M111 | Respiratory signs and symptoms, level 1 | 1.0 | 967 |
| 04M112 | Respiratory signs and symptoms, level 2 | 6.1 | 2,902 |
| 04M113 | Respiratory signs and symptoms, level 3 | 9.3 | 4,736 |

| 04M114 | Respiratory signs and symptoms, level 4 | 14.9 | 10,031 |
| --- | --- | --- | --- |

Table S5 Summary of resource use and costs (at inclusion, at follow-up) in the as randomized population

| **Variable** | **Groups** | | **Difference** | |
| --- | --- | --- | --- | --- |
|  | **Intervention group (n = 726)**  **Mean (±SD) or N (%)** | **Control group**  **(n = 688)**  **Mean (±SD) or N (%)** | **Adjusted**  **ΔCosts or ΔEffects (95% CI)** | **Unadjusted**  **ΔCosts or ΔEffects (95% CI)** |
| **As randomized population** | | | | |
| **Index Admission** | | | | |
| **1. ED visit** | | | | |
| **YEARS score = 0^*^** | 585 (80.6) |  | - | - |
| **CTPA or [V / Q] lung scan done^**^** | 221 (30.4) | 275 (40.0) | - | - |
| \| **2. Hospitalization** \| \| --- \| | | | | |
| **Admitted from the ED** | 157 (21.6) | 178 (25.8) | - | - |
| **Average length of stay index (Days)** | 1.93 | 2.03 | - | - |
| **Re-hospitalization: Follow-up period at 3 months** | | | | |
| **VTE at 3 months** | 3 (0.41)  [0.09 to 1.20] | 6 (0.87)  [0.32 to 1.89] | - 0.49   [97.5% 1-sided CI**,** -∞ to 0.36] | -0.46  [97.5% 1-sided CI, -∞ to 0.45] |
| **Mean cost per patient (€)** | | | | |
| **Emergency Departments** | 116 (±38) | 123 (±45) | - | -7 [95% CI, -12.8; -3.2] |
| **All-cause Index hospitalization** | 178 (±388) | 213 (±374) | - | -35 [95% CI, -72; 10] |
| **Hospitalization admission at 3 months related to PE** | 1.3 (±23) | 16.5 (±198) | - | -15.2 [95% CI, -38; -2.5] |
| **Total costs** | 295 (±404) | 352 (±515) | -44 [95% CI, -84 ; -2] | -57 [95% CI, -106; -9] |

*YEARS score ranges from 0 to 3, 1 point per item: PE is the most likely diagnosis, hemoptysis, and clinical sign of deep vein thrombosis.

** One patient in the control group had CTPA and [V˙ /Q˙] scan.


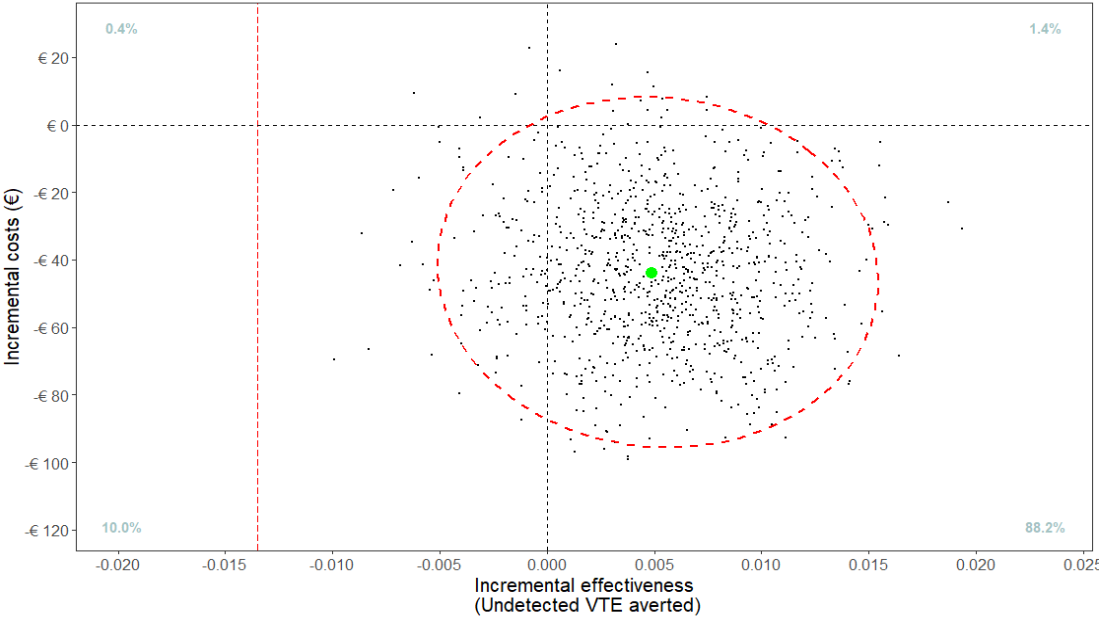


Figure S2: Bootstrap distribution of 1000 ICER in the as randomized population (€ /patient without VTE at three months- diagnostic failure) (The vertical red line indicates the non-inferiority margin)
